# Acute Abdomen in Pregnancy: A Comprehensive Review of Diagnosis and Management

**DOI:** 10.7759/cureus.40679

**Published:** 2023-06-20

**Authors:** Rishi Dhamecha, Sandhya Pajai, Taanvi Bhasin

**Affiliations:** 1 Obstetrics and Gynecology, Jawaharlal Nehru Medical College, Datta Meghe Institute of Higher Education and Research, Wardha, IND

**Keywords:** diagnostic laparoscopy, surgical acute abdomen, ovarian torsion, ultrasound, obstetric causes

## Abstract

An unusual diagnostic and treatment challenge is presented by an acute abdomen during pregnancy. Obstetric factors and other causes unrelated to pregnancy also contribute to acute abdominal discomfort in pregnancy. Due to the changing clinical presentations brought on by the anatomical and physiological changes of pregnancy as well as the hesitation to utilize certain radiological studies out of concern about damaging the fetus, the diagnosis of the acute abdomen during pregnancy can be challenging. Delays in identification and treatment may have negative effects on the mother as well as the fetus.

This review sheds light on the importance of anatomical and physiological considerations, early diagnoses, and understanding the various modalities and etiologies of acute abdomen in pregnancy (AAP). We then move on to discuss the various diagnostic techniques that can help the physician determine the causes and plan well-informed treatment. We examine and contrast different radiographic tests, including X-rays, computed tomography, magnetic resonance imaging, and ultrasound. We also talk about the various roles that these investigational methods can play in the evaluation and treatment throughout the duration of the pregnancy.

The paper additionally addresses how to handle patients who appear with AAP and the different techniques used to treat them, including pre-operative laparoscopy. Before going over some more broad points that might be useful, we eventually dive into some of the more intriguing etiologies relating to AAP, such as isolated tubal torsion and neoplastic complications.

## Introduction and background

The diagnosis and treatment of acute abdomen in pregnancy (AAP) constitute a special difficulty. Any major acute intra-abdominal ailment associated with pain, soreness, and muscle stiffness is referred to as an acute abdomen and should be in consideration for treatment with emergency surgery. It frequently signals a clinical constellation of abdominal symptomatology that may last for hours to days or even weeks. The term is sometimes used interchangeably for any abdominal ailment that necessitates prompt surgical intervention. A daunting diagnostic and therapeutic challenge is presented by the broad array of etiologies and manifestations [1].

Acute abdomen in pregnancy must be considered an emergency and be treated with prompt care and the involvement of a multidisciplinary team. The involvement of a general surgeon, obstetrician and gynecologist, maternal-fetal expert, and radiologist is necessitated by the sheer gravity of the situation and offers the route to a much better outcome than otherwise possible. The approach to a pregnant patient presenting with acute abdomen, as per the definition already discussed above, is to be done in the same manner as required by someone in a non-pregnant state, with some changes.

AAP's diagnosis methodology can be challenging. This is a result of both the static and dynamic metabolic alterations induced by gestation. A low barrier for subjecting the patient to an emergency surgical operation and resistance to using radiological diagnosis tools like computed tomography (CT) scan or X-ray are other contributing factors. It can also be challenging to physically examine the belly while the patient is pregnant. For a prompt and precise identification of possibly life-threatening scenarios, which without it could be dangerous for both mother and unborn child, a methodical approach is required.

## Review

Methods

**Figure 1:**
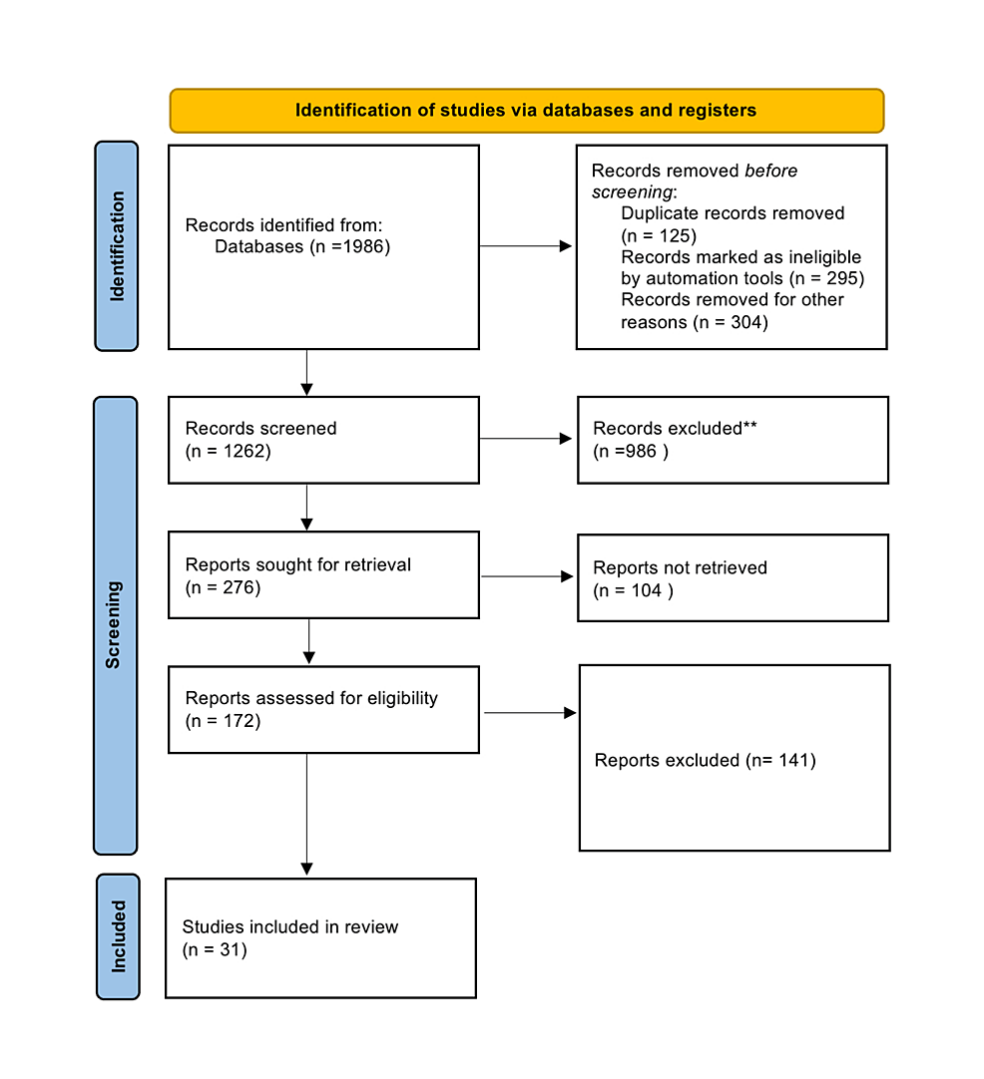
PRISMA flowchart for search PRISMA: Preferred Reporting Items for Systematic Reviews and Meta-Analyses.

The terms "diagnostic laparoscopy," "surgical acute abdomen," "ovarian torsion," "ultrasound," "acute abdomen," and "abdominal pregnancy" were searched for in a database like PubMed. Only results pertaining to the English language were shown. If there was more than one published report from a similar study, the latest one was used. Only review articles that also had original data were taken into account. The PRISMA (Preferred Reporting Items for Systematic Reviews and Meta-Analyses) flowchart for the search is shown in Figure 1.

Anatomical considerations

Around 12 weeks into the pregnancy, the uterus, which is typically a pelvic organ, grows to become an intra-abdominal organ. The uterus can grow during pregnancy from just 70 g to 1110 g, resulting in an intrauterine volume of at least 5 liters. Early in the gestational period, the uterus develops into a thick-walled muscular organ as a result of the hyperplasia and hypertrophy of the muscle fibers. The uterus may be touched near the umbilicus by the 20th week, and the intrinsic growth practically stops [1]. The uterus expands more as a result of mechanical stretching of the muscle fibers and distension caused by the expanding fetus. The uterus reaches the costal edge at 36 weeks. To meet the growing needs, the uterine blood vessels also experience substantial hypertrophy [1].

Physiological considerations

A coordinated interaction of hormones, particularly progesterone, causes physiological changes that affect almost all organ systems and result in a broad shift in the uterine environment. Endocrine, metabolic, circulatory, GI, renal, musculoskeletal, respiratory, and behavioral modifications are among them. Of the expectant women, 50% to 80% may experience GI alterations like delayed stomach emptying, longer intestinal transit time, gastroesophageal reflux, abdominal bloating, nausea, and vomiting. The mechanical compression of the intestines during the third trimester is thought to contribute to constipation, as is the rise in water and salt intake brought on by elevated aldosterone levels [1].

Obstetric causes of acute abdominal pain

Abdominal discomfort during pregnancy may have natural or pathological origins. The round ligament pain brought on by stretching of the round ligament, the discomfort experienced during fetal movement, and painful Braxton-Hicks contractions may be the physiological reasons for stomach pain in pregnancy. A total of 10% to 30% of pregnancies can become complicated by round ligament discomfort, which often happens toward the conclusion of the first and beginning of the second trimester. It is a cramp-like discomfort that is confined to the lower abdomen quadrants and radiates to the groin; it is more frequent in multiparous women. Round ligament discomfort raises concerns since there is a propensity to overdiagnose this benign illness, which can prevent or postpone the discovery of serious pathological factors that pose a life-threatening risk. Ectopic pregnancy rupture, abruption, HELLP (hemolysis, elevated liver enzymes, and low platelet) syndrome, acute fatty liver of pregnancy, and uterine rupture are pathological reasons that can be fatal [2].

Acute abdominal pain in the first trimester of pregnancy

Women who are pregnant and arrive at the emergency department (ED) with nausea or vomiting are likely to have their pregnancy assessed. Despite the prevalence of disease associated with pregnancy, certain patients may have intra-abdominal pathology that necessitates immediate assessment and perhaps surgical intervention [3].

Since some degree of stomach discomfort is practically physiological throughout pregnancy, these women frequently visit the emergency room for a clinical assessment. The most prevalent non-obstetric surgical condition that complicates pregnancy is appendicitis. The clinical evaluation of these individuals is typically complicated by the symptoms common to both these disorders and pregnancy, which make a physical examination (PE) of the abdomen difficult. However, to reduce maternal and particularly fetal mortality, rapid clinical diagnosis and surgical intervention, where appropriate, are required. When a doctor encounters patients who are critically sick or unstable, they must ignore the common reluctance to perform standard radiography study on them. Broad use of surgical and obstetric experts is recommended if the diagnosis is unclear [4].

Emergent MRI for acute abdominal pain in pregnancy

Ultrasonography (USG) is not always successful in identifying the reasons for acute abdominal discomfort, and magnetic resonance imaging (MRI) is becoming more and more popular in this situation. Pregnancy-related acute abdominal discomfort is still a clinically difficult presentation that frequently necessitates imaging. Rapid and precise imaging diagnosis is required due to the risk of morbidity and death to both mother and fetus.

In case ultrasound comes up with inconclusive results, MRI should be taken into consideration as a reliable inquiry to determine the reason for acute abdominal and pelvic discomfort during pregnancy [5].

The diagnosis of acute abdominal discomfort in pregnant individuals is challenging. Because there are many potential causes of abdominal discomfort during pregnancy, including diseases of various intra-abdominal or intrapelvic tissues, there is a wide range of differential diagnoses that can be made. The anatomical and physiological changes that take place during pregnancy make it challenging to pinpoint the location of illness. The potential for ionizing radiation to damage the fetus restricts the use of standard radiography imaging [6].

In the assessment of sudden abdominal discomfort, CT is often used. Excellent anatomical information is provided by CT, but the fetus receives a sizable dose of ionizing radiation, making this approach unfavorable. When a pregnant patient complains of abdominal pain, sonography is frequently the first imaging method employed. Sonography is a safe and adaptable imaging procedure. However, internal organs may be dislocated and difficult to see on sonography when a gravid uterus is present [6].

The diagnosis of a pregnant woman with severe pelvic or abdominal discomfort presents a special problem. MRI is a great option for evaluating these individuals because of its inherent safety and accuracy in identifying abdominal and pelvic illnesses in pregnant patients [6].

Diagnostic and therapeutic problems are presented by acute abdominal discomfort during pregnancy. Due to the potential for teratogenic and carcinogenic effects of X-rays, conventional imaging procedures must be modified. The major imaging study of the pregnant abdomen is still ultrasound. It has been demonstrated that MRI is helpful in the diagnosis of obstetric and gynecological issues during pregnancy, as well as in the case of an acute abdomen. Some of the shortcomings of USG are resolved by MRI, particularly the size of the gravid uterus [7].

It is important to take precautions to reduce any potential dangers associated with MRI for the fetus by avoiding contrast chemicals. For imaging the suspected disorders, recommendations based on the most recent research and the working group's cumulative clinico-radiological expertise of the European Society of Urogenital Radiology (ESUR) are utilized and considered [7].

Ultrasound is a useful first-line diagnostic technique for expecting mothers who are experiencing abdominal discomfort. The ultrasonic examination in the same room as the clinical evaluation speeds up the process of making a diagnosis. The hazards for mother and child may be rapidly identified and the required diagnostic and treatment actions can be performed by using a systematic approach and classifying sources of pain into pregnancy-related sources of pain and pregnancy-unrelated sources of pain. Experienced sonographers are required, and top-notch ultrasound equipment must be on hand [8].

In pregnant women presenting with an acute abdomen, sonography continues to be the primary line of imaging. Based on the sonographic results, patients may be categorized to undergo further imaging [9].

MRI's effectiveness in the diagnosis and triage of pregnant women presenting with severe abdominal or pelvic pain has been evaluated retrospectively. For the diagnosis of acute appendicitis and the exclusion of illnesses needing surgical or interventional therapy, MRI is a superb modality. Consequently, MRI is helpful for triaging pregnant women who are experiencing severe pelvic and abdominal discomfort [10].

Pregnancy-related abdominal discomfort is very typical; however, it is uncommon for surgical pathology like acute appendicitis to be the source of this pain. In addition to the history and PE, diagnostic techniques including MRI and USG are employed. The ultrasound provides minimal benefit and patients should move on to MRI in expectant women who had severe abdominal discomfort and a positive PE strongly indicative of surgical pathology [11].

Approach to the acute abdomen in pregnancy

The way that abdominal discomfort manifests during pregnancy might vary depending on a number of physiologic factors. When assessing a patient who is pregnant and experiencing abdominal pain, a strong index of suspicion must be applied. Pregnancy-related general anesthesia is regarded as safe. Individualization of tocolytics and intraoperative monitoring is necessary. When feasible, laparoscopic surgery should be done during the second trimester since it seems to be just as safe as laparotomy. Diagnostic imaging should not be withheld from a pregnant patient if it is indicated [12].

Pregnancy appears to be safe for both cholecystectomy and appendectomy. With the increased use of first-trimester USG, there may be a rise in the reported incidence of adnexal masses and fibroids in pregnancy. In most situations, conservative care combined with postpartum surgery seems prudent [12].

Preoperative Laparoscopy in Diagnosis of Acute Abdominal Pain

At the Peter Bent Brigham Hospital in Boston, Massachusetts, in 1973, 56 patients in one of the three general surgical departments who were deemed to need hospital admission for severe abdominal discomfort were split into two groups. Whether or not the prescribing doctor believed a firm diagnosis could be made based only on clinical evidence determined this divide. Without a preoperative laparoscopy, 27 patients who were believed to have a certain diagnosis had laparotomies; at the time of the procedure, six of these patients (22%) had no operable lesions. A total of 29 more individuals needed inpatient observation due to severe abdominal discomfort. Clinically, these patients could not be given an exact diagnosis, and many of them would have previously needed exploratory laparotomies [13].

These 29 patients underwent laparoscopy, and all but one (4%) had intra-abdominal illness needing operational intervention definitively confirmed as either present or absent. In 18 patients who had laparoscopy but did not need a laparotomy, a diagnosis was obtained; nevertheless, in 11 patients, a laparotomy was necessary due to their condition. There were no side effects from the laparoscopy. When laparoscopy rather than an exploratory laparotomy revealed that acute stomach pain was caused by a disease not needing surgical intervention, the difference in the median length of stay and hospital costs led to a savings of one and a half days in the hospital and a considerable amount of money [13].

Acute abdomen in early pregnancy due to ovarian torsion following successful in vitro fertilization treatment

Acute abdominal pain from ovarian torsion needs immediate treatment. Pregnancy seldom causes ovarian torsion. Nevertheless, ovarian hyperstimulation during in vitro fertilization (IVF) procedures might enlarge the ovaries and cause adnexal torsion [14].

A risk factor for adnexal torsion during IVF-embryo transfer therapy is ovarian hyperstimulation. The only approach to save the ovary and maintain the pregnancy is by early detection and rapid surgical intervention. Once the diagnosis is established, laparoscopic surgery should be promoted because it does not damage the fetus during early pregnancy. Delaying surgery might result in a major infection and endanger both the mother's and the fetus' lives [14].

Isolated tubal torsion as a cause of acute abdomen in pregnancy

Only a very tiny percentage of instances of acute abdomen in pregnancy is caused by solitary fallopian tube twisting, which is a rare cause of adnexal torsion. Both genital and non-genital causes of these disorders, whether they occur during pregnancy or outside of pregnancy, are recognized, and in the majority of instances, predisposing factors may be identified. An isolated tubal-paratubal cyst torsion should be taken into consideration in instances of the acute abdomen during pregnancy, with specific Doppler flow USG indicative of normal ovaries and a pelvic cyst, and the proper ovary-sparing surgical therapy should be anticipated [15].

When there is an axial rotation of more than 40%, uterine torsion is present. The existence of fibroids and the use of an external cephalic version to rectify a malpresentation may be the causes of uterine torsion. Uterine torsion symptoms might include discomfort, shock, and bowel or urine issues. Torsion of the uterus can result in vasovagal shock in the mother and consequent fetal suffocation. Conservative measures, such as bed rest, analgesia, and changing the mother's position, as well as surgical measures, such as laparoscopic uterine detorsion and hoping to continue the pregnancy if the fetus is preterm, or performing a C-section after detorsion in a viable fetus, are among the management options available for uterine torsion. Detorsion can be done in cases with ovarian/adnexal torsion without increasing the risk of problems for the mother or the fetus. Excision must be done if the cyst is gangrenous [1].

The surgical technique in gynecology has been significantly impacted by laparoscopy and pelviscopy. Today, laparoscopic surgery may be used to treat most acute abdomens. Classic laparotomy is necessary for some of the disorders covered. The preservation of a woman's ability to procreate has a significant influence on her well-being [16].

Although MRI is more frequently utilized in this case to minimize radiation exposure, computed tomography can also be employed prospectively in some individuals to further analyze abnormalities previously discovered in sonography [17].

Gynecological neoplasms with acute presentation

Gynecological neoplasms of various forms can occasionally present abruptly in patients, and the diagnosis will not be suspected clinically until a CT scan is done since the patient's abdominal and/or pelvic discomfort is otherwise vague. Urinary frequency, early satiety, and abdominal pressure from the mass effect might all be concurrent symptoms. These include the previously mentioned benign underlying ovarian neoplasms, such as cysts and teratomas, which can present with torsion (frankly, malignant ovarian tumors are more frequently fixed and, therefore, rarely present with torsion); additionally, these include the occasional ovarian carcinoma, uterine carcinoma, or gestational trophoblastic neoplasm, which presents acutely for a variety of reasons (i.e., hemorrhage, abrupt onset of ascites, and necrosis) [17-21].

General considerations

As a result of primary illness, pathologic processes on some organs, or accidents, the syndrome of acute abdomen in gynecology and obstetrics can arise owing to inflammatory, ischemic-obstructive, and hemorrhagic intra-abdominal etiologies (genital, gastrointestinal, urinary, vascular, neurologic, and musculoskeletal systems), along with neoplastic etiologies [18,22-27].

Diagnostic and therapeutic problems are presented by acute abdominal discomfort during pregnancy. Due to its accessibility, mobility, and absence of ionizing radiation, USG continues to be the most common imaging study of the pregnant abdomen. It has been demonstrated that MRI is helpful in the diagnosis of gynecologic and obstetric issues as well as in the case of an acute abdomen during pregnancy. When USG is not definitive, MRI is frequently employed. When a person's life is in danger or when a quick diagnosis is needed for catastrophic injuries, CT is the investigation of choice [19,28-31].

Although pregnancy masks the clinical presentation and increases the likelihood of an acute abdomen, early surgical surgery improves the perinatal prognosis and reduced maternal morbidity. Tocolytics may cause negative side effects and have no influence on the health of the fetus [20].

## Conclusions

AAP presents with a constellation of different manifestations that may point toward vague and nonspecific findings and diagnoses. In our review of the literature, after taking into consideration all the anatomical and physiological factors relating to the pregnant state, we move on to discuss the various etiologies and modalities that can precipitate this condition. We discuss the various physiological as well as pathological scenarios that may bring about these changes in pregnancy. We further discuss the possibilities of AAP occurring in the first trimester, and its implications.

Furthermore, we move on to the various diagnostic modalities that may assist the clinician in identifying the causes and planning well-guided management of the condition. We take a look at and draw comparisons between various radiological investigations such as X-ray, CT, MRI, and USG. We also discuss how at different points of time in the course of the pregnancy, these investigational modalities can play different roles in the diagnosis and management. The article also discusses the approach to a patient presenting with AAP and the various procedures deployed for their management such as pre-operative laparoscopy. We finally delve into some of the more interesting etiologies pertaining to AAP, such as isolated tubal torsion and neoplastic complications, and discuss them at some length, before discussing some general considerations that may be helpful.
